# Light-controllable dithienylethene-modified cyclic peptides: photoswitching the in vivo toxicity in zebrafish embryos

**DOI:** 10.3762/bjoc.16.6

**Published:** 2020-01-07

**Authors:** Sergii Afonin, Oleg Babii, Aline Reuter, Volker Middel, Masanari Takamiya, Uwe Strähle, Igor V Komarov, Anne S Ulrich

**Affiliations:** 1 Institute of Biological Interfaces (IBG-2), Karlsruhe Institute of Technology (KIT), POB 3640, 76021 Karlsruhe, Germany; 2 Institute of Organic Chemistry (IOC), KIT, Fritz-Haber-Weg 6, 76131 Karlsruhe, Germany; 3 Institute of Toxicology and Genetics (ITG), KIT, POB 3640, 76021 Karlsruhe, Germany; 4 Taras Shevchenko National University of Kyiv, vul. Volodymyrska 60, 1601 Kyiv, Ukraine; 5 Lumobiotics GmbH, Auerstr. 2, 76227 Karlsruhe, Germany

**Keywords:** diarylethene photoswitch, gramicidin S, membrane-active peptides, photopharmacology, zebrafish embryotoxicity model

## Abstract

This study evaluates the embryotoxicity of dithienylethene-modified peptides upon photoswitching, using 19 analogues based on the β-hairpin scaffold of the natural membranolytic peptide gramicidin S. We established an in vivo assay in two variations (with ex vivo and in situ photoisomerization), using larvae of the model organism *Danio rerio,* and determined the toxicities of the peptides in terms of 50% lethal doses (LD_50_). This study allowed us to: (i) demonstrate the feasibility of evaluating peptide toxicity with *D. rerio* larvae at 3–4 days post fertilization, (ii) determine the phototherapeutic safety windows for all peptides, (iii) demonstrate photoswitching of the whole-body toxicity for the dithienylethene-modified peptides in vivo, (iv) re-analyze previous structure–toxicity relationship data, and (v) select promising candidates for potential clinical development.

## Introduction

Biologically-active peptides as a class of chemotherapeutic compounds are uniquely positioned between traditional small organic molecule drugs and high-molecular weight biologics [1–2]. Since recent breakthroughs in peptide synthesis technology [3–4] have enabled peptide production at industrial scales, the exploration of therapeutic peptides as potential drugs is rapidly developing [4–6]. It has been shown that peptide drugs are less immunogenic than biologics and can hit the “undruggable” space of molecular targets [2,7–8]. As a result, the market for peptide drugs (e.g., hormones, receptor antagonists, anticancer, or antibiotic agents) grows faster than that of many other chemotherapeutics [9]. Significant general disadvantages of peptides as pharmacological agents are their poor oral bioavailability, low plasma stability in vivo, limited understanding of their mechanisms of action, and high in vivo toxicity. The former two drawbacks are being adequately resolved [9] (e.g., by modifying the peptide backbone [10], macrocyclization [11], or by use of unnatural amino acids [12]), and mechanistic understanding of the relevant molecular mechanisms is gradually improving [13]. However, the safety consideration still poses a considerable challenge. Hence, the understanding and decreasing of the in vivo toxicity of peptides is of paramount importance for their development as drugs.

In recent years, photopharmacology [14–16] has emerged as a successful approach to enhance spatiotemporal selectivity of chemotherapeutics, decreasing the overall toxicity and increasing the safety of drugs. Various compound classes are currently being explored as photopharmacology agents [16–21], including peptides [19–21]. The idea behind photopharmacology is based on the design and use of drugs containing a reversibly photoisomerizable fragment (“molecular photoswitch”) as part of their structure. Importantly, the photoisomerizable fragment should – in one of its photoforms – destroy or mask the pharmacophore (the drug is “switched OFF”), whereas the other photoform should restore the biological activity of the active element (the drug is “switched ON”). Correspondingly, an inactive drug (the OFF photoform) can be safely administered, and subsequently it can be locally activated for the therapy (converted to the ON photoform) by applying light of a specific wavelength, precisely at the desired site of action. Spatiotemporal resolution of such light-mediated drug delivery is limited mainly by the technical characteristics of the medical devices used for light application and by the light propagation in tissues.

**Figure 1:**
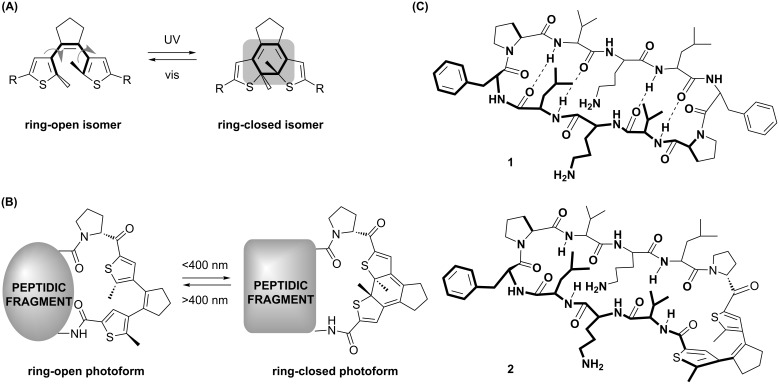
DAE photoswitch and photoswitchable peptides explored in this study. (A) The reversible photoisomerization of the dithienylethene photoswitch core. (B) Photoconversion between ring-open and ring-closed photoforms of DAE-derived peptides. (C) Structures of the non-photoswitchable cytotoxic peptide GS (**1**), and one of our first photoswitchable prototypes **2** in the ring-open photoform.

Among several known photoswitches [18] that are being used in peptides [19–23], diarylethenes (DAE) have increasingly attracted attention in recent years [22–23]. Photoswitchable DAE-derived molecules offer several advantages for medical applications, as their photoforms are thermally irreversible and highly fatigue resistant [24–26]. Light-induced reversible pericyclic reactions toggle the structure of DAE between a flexible ring-open isomer and a planar rigidified ring-closed form (Figure 1A). Irradiation of the ring-open isomer with UV light (<400 nm) generates the ring-closed isomer, while irradiation with visible light (>400 nm) converts the ring-closed isomer back to the ring-open photoform (Figure 1B), thereby affecting the structure and flexibility of the immediate molecular surrounding.

We have constructed a number of DAE-containing peptides by incorporating a DAE photoswitch into the backbone of gramicidin S (**1**, GS), which is an intrinsically biostable cyclic peptide, as exemplified by one of our first prototypes compound **2** (Figure 1C). This natural antimicrobial agent has an amphipathic structure, whose functional mechanism is attributed to the permeabilization of bacterial membranes, with considerable side-effects also on eukaryotic cell membranes [27–28]. Compared to the ring-closed (deactivated, OFF) photoform, the ring-open (active, ON) isomer of **2** indeed displayed a much stronger in vitro toxicity against several tumor cell lines [29–30]. Using the photoswitchable analogue **2** in an allograft mouse model, the first in vivo photopharmacology application for a DAE-derived peptide as an anticancer agent has been demonstrated [29]. In order to optimize **2**, we have recently performed a structure–activity relationship (SAR) study using a library of photoswitchable derivatives of **2** [30]. The library contained 29 compounds grouped into several series: with natural amino acid mutations affecting the amphipathicity (series i), with point mutations that modulate polarity and conformational stability of the β-structural elements (series ii), with backbone *N*-alkylation and side-chain hydroxylation modifications (series iii), with macrocycle ring-size variations (series iv), and with macrocycle homodimerization (series v). We systematically screened the ring-open and ring-closed photoforms of all 29 compounds in vitro, using a range of cellular toxicity assays (against Gram-positive bacteria, Gram-negative bacteria, HeLa cells, and human erythrocytes) and were able to rationalize the specific impact of our modifications onto cell selectivity indices. Though all compounds demonstrated distinctly different cell toxicities in the ring-open and the ring-closed photoforms, we noticed that there was generally a poor correlation between the antibacterial activity on the one side, and the cytotoxicity against HeLa cells on the other side, and erythrocytes as a third scenario. Different compounds could thus be regarded as potential leads for chemotherapy of either infectious diseases caused by Gram-positive or -negative bacteria, or for anticancer applications. We hypothesized that the lack of correlation between cell types might not only reflect different mechanisms of killing (possibly even within any pair of isomers), but that it may also be due to unique differences of erythrocytes compared to other somatic cells. Although easily measured, hemolytic activity gives only a rough estimate of toxicity for peptide therapeutics. Erythrocytes are terminally differentiated non-adherent organelle-free cells, densely packed with oxygen-carrying proteins; their homeostasis and pharmacokinetics are highly specialized, and many molecular targets are absent in them [31].

Aiming at applications of photoisomerizable drugs in human healthcare, investigators cannot be limited to in vitro toxicity assays. Furthermore, preclinical drug development programs specifically require the inclusion of in vivo toxicity studies using vertebrate animal models. (For example, toxicity studies of anticancer chemotherapeutics are requested by the International Council for Harmonisation of Technical Requirements for Pharmaceuticals for Human Use (ICH) to include at least two mammalian species, whereby one of them must be a non-rodent [32]). Comprehensive in vivo toxicity studies are obviously lengthy, significantly more expensive than in vitro assays, and are hampered by strict bioethics regulations. We therefore looked for an alternative toxicity assay that would be as technically simple as hemolysis, and at the same time would make the data more relevant to human toxicity. Hence, we selected the zebrafish embryotoxicity assay as a potential compromise. Due to their small size, cheap husbandry and maintenance, fast embryogenesis, extracorporeal development, known genome and accessibility of several thousand transgenic lines [33], zebrafish (*Danio rerio*) is an excellent model for developmental biology and phenotypic genetics [34]. The zebrafish species are attractive to utilize them in toxicity studies due to several reasons. Optimum maintenance and breeding conditions for *D. rerio* are well described in the literature [35], as well as complete details of its embryogenesis [34]. Furthermore, the vertebrate body plan of the zebrafish is, in its basic structure, similar to mammals [36–37], and up to 80% of the known human drug targets are present in the *D. rerio* genome [38]. Since 2005, a zebrafish embryotoxicity test is used as a standardized ISO assay for sewage water testing in Germany [39–40]. Due to its manifold advantages, zebrafish larvae could be used as a cost-effective vertebrate animal model, yet sophisticated enough for pharmacological toxicity evaluations, especially for preclinical drug candidate screenings. However, to the best of our knowledge, such applications for therapeutic peptides are still sparse [41–45]. Notably, zebrafish embryos are transparent, which makes them ideal for in vivo manipulation of photosensitive compounds [46]. This has been advantageously used by other authors in studies of azobenzene-containing photoswitchable bioactive agents [47–50]. Finally, according to the recent edition of EU directive 2010/63/EU, zebrafish embryos of up to 5 days post fertilization (dpf), as the larvae are still feeding on the yolk [51], are excluded from the legislation governing animal testing, i.e., experiments do not require ethical approval.

In this study, we selected 19 photoswitchable DAE-modified cytotoxic peptides from our previous SAR evaluation [30] and established with them the *D. rerio* embryotoxicity assay. For each photoswitchable peptide, we determined the lethal dose of both photochromic forms, using two assay variations, and these in vivo toxicity values could then be compared with the known in vitro cytotoxicities.

## Results and Discussion

All peptides (Table 1) were synthesized as was previously described [30] and initially handled as ring-open photoforms under ambient (visual, vis) light conditions. Each of the ring-open photoforms was converted into the ring-closed photoforms by UV irradiation in a solution of denaturing agents, reproducing previously documented results [29–30,52]. All studied compounds were prepared in >95% purity using preparative-scale high-performance liquid chromatography (HPLC).

**Table 1:** Nomenclature, sequences, molecular masses of the peptides synthesized and explored in this study. The photoswitchable peptides are grouped in series (i–v) according to their original design [30].

Peptide	Linear sequence^a^	Molecular mass, *m*/*z*	
		calculated	measured

**1**	fPVOLfPVOL	1141.4	1141.2
**2**	DAE-VOLfPVOL	1280.7	1280.1

analogues with point mutations affecting amphipathicity (series i)			

**3**	DAE-VOLfPVOA	1238.6	1238.1
**4**	DAE-AOLfPVOA	1210.6	1210.1
**5**	DAE-OOLfPVOL	1295.7	1295.1

mutations affecting polarity and stability of β-structures (series ii)			

**6**	DAE-TOVfPVOV	1254.6	1254.1
**7**	DAE-Asn^iPr^-OVfPVOV	1309.7	1309.3
**8**	DAE-Dab^iBu^-OVfPVOV	1323.7	1323.3
**9**	DAE-IOLpPIOL	1258.7	1258.5

analogues with *N*-alkylation and hydroxylation modifications (series iii)			

**10**	DAE-IOIf-Leu^N-Me^-LOI	1338.8	1338.8
**11**	DAE-IOLf-Leu^N-Bu^-IOL	1380.9	1380.4
**12**	DAE-Leu^OH^-OVfPVOV	1282.7	1282.3
**13**	DAE-VOVfP-Leu^OH^-OV	1282.7	1282.4
**14**	DAE-VOVfPVO-Leu^OH^	1282.7	1282.4
**15**	DAE-Leu^OH^-OVfP-Leu^OH^-OV	1312.7	1312.4

analogues with extended macrocycles (series iv)			

**16**	DAE-VKLKVfPLKVKL	1777.4	1776.2
**17**	DAE-VKLKVfPLkVKV	1763.3	1762.2
**18**	DAE-VKLKVfPKLVKV	1763.3	1762.2

“click”-chemistry connected homodimers (series v)			

**19**	DAE-Leu^OH^-OLf-OrnN_3_^N-Me^-VOL^b^	2773.5^c^	2773.1^c^
**20**	DAE-OrnN_3_-OLfPVOL^b^	2681.4^c^	2681.0^c^

^a^Canonical amino acids and ornithine (designated O) are given in one-letter code. Lower case letters are for amino acids with ᴅ-configuration. Non-canonical amino acids are presented by three-letter code. Superscript indices *N*-Me and *N*-Bu, next to a three-letter abbreviation, indicate *N*-methylation and *N*-butylation, respectively. Leu^OH^ = (2*S*,3*R*)-β-hydroxyleucine; Asn^iPr^ = *N*^γ^-isopropylasparagine; Dab^iBu^ = *N*^γ^-isobutyryldiaminobutyric acid; OrnN_3_ = L-δ-azidoornithine; DAE photoswitching fragment = 4-(2-(5-((ʟ-prolyl-2-methylthiophene-3-yl)cyclopent-1-en-1-yl)-5-methylthiophene-2-carboxyl); ^b^monomers (as listed here) were dimerized through azides of the azidoornithines via propargyl ether by means of Cu(I)-catalyzed “click” reaction [29]; ^c^molecular masses are for final ether-conjugated dimers.

**Figure 2:**
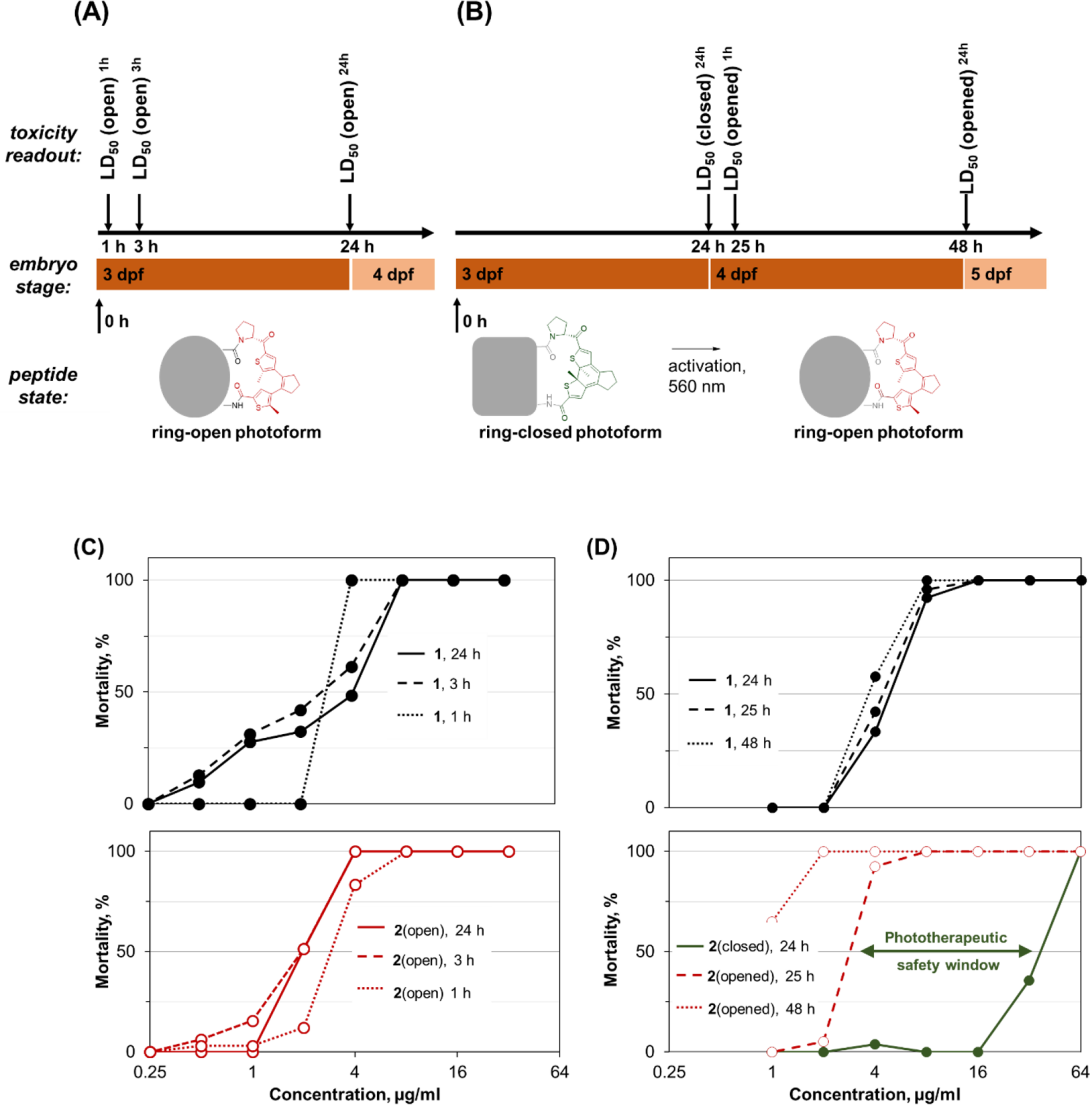
Two versions of the *D. rerio* embryotoxicity assay for DAE-modified peptides: timelines, peptide photostates, ages of larvae, and definitions of the measured characteristics. (A) Direct application of the non-photoswitchable parent GS **1** or of the ring-open photoform of our prototype **2_,_** and the corresponding killing curves for **1** (C, top, black) and **2** (C, bottom, red). (B) “Photoactivation” assay: initial application of the ring-closed photoform, incubation in darkness, followed by in situ photoconversion into the ring-open(ed) photoform, with the corresponding killing curves for **1** (D, top) and **2** (D, bottom, green for ring-closed, red for ring-open isomers). The latter panel illustrates the “phototherapeutic safety window” (green arrow) as the difference in toxicity between the ring-closed and the ring-open isomers upon photoactivation.

To establish the assay for screening the photoswitchable peptides, we first studied the toxicity of our prototype **2** by varying the assay conditions. The parent peptide GS **1** was used as a control. Although the parent **1** does not contain a photoswitchable moiety, it was treated throughout the assay in the same fashion as **2**, in order to account for both (i) the light application effects as well as (ii) the expected [53] developmental changes of the embryos during the experiment. We measured the doses causing the death of 50% embryos (LD_50_). We tested two different assay variations: in one version, the LD_50_(open) was determined after direct application of **1** or of the ring-open form of **2**. In the other variant, the photoswitchable peptide was applied in the ring-closed photoform in the darkness, its LD_50_(closed) was recorded, and then in situ illumination (“photoactivation”) was applied to switch into the ring-open photoform and obtain the respective LD_50_(opened) values (see Figure 2 for the study design and the killing curves).

Each time, 12–15 or 18–20 zebrafish embryos at 3 dpf were treated by applying the corresponding peptide dissolved in dimethyl sulfoxide (final dimethyl sulfoxide concentration was 0.2%). Double-concentrated peptide stock solutions (64–512 µg/mL) were prepared by weighing lyophilized peptides, dissolving in 10% dimethyl sulfoxide and constructing two-fold dilution series with ten–eleven dilution steps. In the photoconversion experiments, in order to prevent spontaneous photoswitching of the ring-closed peptides by vis light, zebrafish embryos and peptides were kept in the dark before the first readout, and the vials were sealed to avoid evaporation of the embryonic medium (E3) at prolonged incubation times. After the first screen, without exchanging any solution, the vials with embryos and ring-closed photoforms were exposed to intense illumination for 10 min (LUMATEC Superlite 410 light source, λ = 570 nm, irradiance approximately 20 mW/cm^2^). Mortality was then determined under ambient light conditions at 1 h and 24 h after the illumination procedure.

As shown in Figure 2C, the control **1** (non-switchable parent) and the ring-open isomer of our first prototype **2** displayed very similar LD_50_ values upon direct application. After 24 h, 50% of embryos had died at a concentration of 2.4 ± 0.2 µg/mL GS **1** and 2.1 ± 0.3 µg/mL ring-open **2**. With increasing incubation time, lower concentrations were expected to achieve comparable mortality; however, the difference between 1 h and 24 h incubation was low for both peptides (Table 2).

**Table 2:** Toxicities and LD_50_(closed)^24h^/LD_50_(open)^24h^ ratios for the photoswitchable peptides. The peptides are grouped in series (i–v) according to their original design in [30].

Peptide	Toxicities at different conditions, µg/mL ± STD^a^						LD_50_(closed)^24h^/LD_50_(open)^24h^ ratio

	LD_50_ (open)^1h^	LD_50_ (open)^3h^	LD_50_ (open)^24h^	LD_50_ (closed)^24h^	LD_50_ (opened)^1h^	LD_50_ (opened)^24h^	

**1**	2.8 ± 2.7	2.0 ± 3.0	2.4 ± 0.2	–	–	–	–
**2**	2.7 ± 1.0	1.7 ± 1.1	2.1 ± 0.3	34.2 ± 12.2	3.3 ± 0.7	0.7 ± 1.3	16.3

analogues with point mutations affecting amphipathicity (series i)							

**3**	9.3 ± 7.8	4.8 ± 3.9	4.7 ± 0.1	31.1 ± 4.2	9.5 ± 1.3	6.2 ± 0.6	6.6
**4**	69.8 ± 10.5	40.6 ± 11.2	19.0 ± 1.3	197.7^b^	51.0 ± 7.7	12.9 ± 1.9	10.4
**5**	4.8 ± 2.2	2.8 ± 1.9	3.3 ± 0.7	8.1 ± 1.0	8.1 ± 1.0	5.3 ± 0.5	2.5

mutations affecting polarity and stability of β-structures (series ii)							

**6**	30.8 ± 15.0	13.2 ± 13.3	8.6 ± 0.0	196.9^b^	20.6 ± 1.7	6.3 ± 2.3	22.9
**7**	15.9 ± 4.3	8.7 ± 9.1	5.8 ± 0.4	266.4^b^	18.2 ± 4.6	3.0 ± 2.5	45.9
**8**	12.0 ± 0.8	5.7 ± 0.4	3.9 ± 1.5	472.9^b^	9.6 ± 5.6	3.0 ± 2.7	121.2
**9**	2.0 ± 1.1	2.1 ± 0.8	1.6 ± 0.3	9.1 ± 2.7	2.2 ± 0.5	0.8 ± 0.1	5.7

analogues with *N*-alkylation and hydroxylation modifications (series iii)							

**10**	1.3 ± 1.5	1.0 ± 0.6	1.0 ± 0.6	9.5 ± 0.1	2.2 ± 1.0	1.3 ± 1.5	9.5
**11**	3.0 ± 1.8	2.4 ± 0.7	1.6 ± 0.1	4.4 ± 0.2	4.0 ± 0.3	3.0 ± 1.0	2.8
**12**	9.2 ± 1.1	6.7 ± 0.4	3.6 ± 0.5	77.0 ± 19.4	6.2 ± 0.0	2.1 ± 1.0	21.4
**13**	4.8 ± 3.8	4.3 ± 3.8	2.2 ± 1.0	45.0^b^	6.4 ± 0.4	3.4 ± 0.5	20.5
**14**	8.1 ± 2.0	6.2 ± 0.7	2.0 ± 0.0	50.4 ± 27.2	9.1 ± 4.2	4.6 ± 2.0	25.2
**15**	36.5 ± 2.1	14.1 ± 6.0	7.2 ± 3.8	56.1 ± 12.2	22.9 ± 0.2	7.2 ± 0.7	7.8

analogues with extended macrocycles (series iv)							

**16**	3.1 ± 1.0		1.6 ± 0.0	6.0 ± 0.1	5.6 ± 0.5	5.2 ± 0.5	3.8
**17**	10.5 ± 5.8		3.2 ± 0.2	9.9 ± 0.1	9.9 ± 0.1	9.4 ± 0.7	3.1
**18**	0.9 ± 0.4		0.4 ± 0.2	12.3 ± 1.5	12.3 ± 1.5	12.3 ± 1.5	30.8

“click”-chemistry connected homodimers (series v)							

**19**	2.7 ± 1.7		1.2 ± 0.3	4.8 ± 0.3	4.7 ± 0.5	4.3 ± 1.3	4.0
**20**	3.7 ± 1.9		1.5 ± 0.0	10.7 ± 0.1	8.4 ± 3.3	3.5 ± 0.6	7.1

^a^STD = standard deviation; ^b^STD was not calculated, the measurement was performed once.

The photoactivation assay, in which two photoisomers of **2** were tested, shows the anticipated difference between the non-photoswitchable **1** and the photoswitchable **2** (Figure 2D and Table 2). For **1**, the dose-dependent mortality before and after illumination was very similar, with only marginal deviation caused by prolonged incubation time. In contrast, **2** displayed an about 16-fold decrease in the LD_50_ value after in situ photoactivation. This difference indicates that the ring-open isomer of **2** indeed possesses a higher toxicity than the ring-closed isomer, and it demonstrates in vivo photoswitching of the whole-body toxicity.

Since the determined LD_50_ values were practically identical in 3 independent experiments, both assays were used to evaluate the entire library of 19 photoswitchable peptides (see Table 2). Overall, the two assay versions gave comparable LD_50_ values for each of the tested peptide, namely LD_50_(open)^1h^ vs LD_50_(opened)^1h^, and LD_50_(open)^24h^ vs LD_50_(opened)^24h^. An exception was noted for the elongated analogues **16**–**18**, where the light-generated ring-opened forms did not restore the activity levels observed in the direct application experiment. This behavior could be explained by a decreased proteolytic stability of these lysine-rich analogues. For the remaining 16 photoswitchable peptides, only minor differences in LD_50_ values between the two types of assays were found. These slight discrepancies are attributed to the different developmental stages of the embryos in the two assays. In the direct activation assay the zebrafish embryos were at 3 dpf, whereas in the photoactivation assay they were at 4 dpf when the ring-closed isomer was converted into the ring-open isomer and subsequently measured at 5 dpf. Also, shielding the embryos for 24 h from light exposure might affect embryogenesis and sensitivity of the larvae [53]. Furthermore, the uptake and accumulation of the ring-closed and ring-open isomer could be different in each case.

Due to photochemical properties of DAE, a realistic DAE-derived in vivo photopharmacology agent should have a low toxicity in the ring-closed form and a high activity in the ring-open form [29–30]. Therefore, analysis relating (undesired) toxicity of the closed form to the (desired) activity of the open form is crucial for the drug candidate assessment. To compare results from different assays and to visualize activity relations of the two photoforms, phototherapeutic indices (e.g., HC_50_(closed)/IC_50_(open) and correlation analysis scatter plots are convenient approaches [30]. Analyzing our data, it was interesting to see that the structure–toxicity relationships turned out to be more complicated than it had initially been anticipated and implemented in the original design of the peptide series. Nonetheless, except for the peptides in series iv (**16**–**18**, with increased charge densities) and one of the homodimers (**19**), the difference in activity between the ring-open and the ring-closed photoforms was significant. In all cases, the ring-open states were more active than the corresponding ring-closed photoforms. For approximately half of the peptides (**2**, **7**, **8**, **9**–**13**, and **20**), the ring-open photoforms were even more toxic than the non-switchable parent compound **1**. For our prototype peptide **2**, the ratio LD_50_(closed)^24h^/LD_50_(open)^24h^ was equal to 16.3. The largest drop in toxicity was observed for the inactive forms of peptides **4**, **6**, **7** and **8** (LD_50_(closed)^24^ ≥ 200 µg/mL), followed by the analogs with hydroxyleucine side chains **12**, **13**, **14**, and **15** (with values in the range of 50–80 µg/mL). This trend, naturally, translates into the safety windows being the largest for these compounds. Peptides **7**, **8**, and – surprisingly – **18**, significantly surpassed the safety window of the original prototype **2**.

The data in Table 2 reveals several structure–toxicity correlations. Both of the tested dimers (**19**, **20**) showed relatively inefficient photoactivation. Given that **19** and **20** were observed to photoisomerize readily between the ring-closed and ring-opened isomers, we can presume that homodimerization may affect the way in which the peptides interact with their molecular targets, or it may compromise their long-term biostability. Likewise, an elongation of the β-sheet core and an increase in cationic charge density (peptides **5**, **15**–**17**) was seen to decrease the photoactivation efficiency in vivo. The corresponding LD_50_ values indicate that an uncharged polar amino acid residue next to the DAE (compounds **6**, **7**, **8**) and the presence of hydroxyleucine residues (**12**, **13**, **14**) improve the photoactivation efficiency compared to compound **2**. Even though this prototype **2** already had quite a large phototherapeutic safety window (the ratio LD_50_(closed)^24h^/LD_50_(open)^24h^ is 16.3), the new mutants showed even higher safety, the best being peptide **8** (with a ratio LD_50_(closed)^24h^/LD_50_(open)^24h^ of 121).

**Figure 3:**
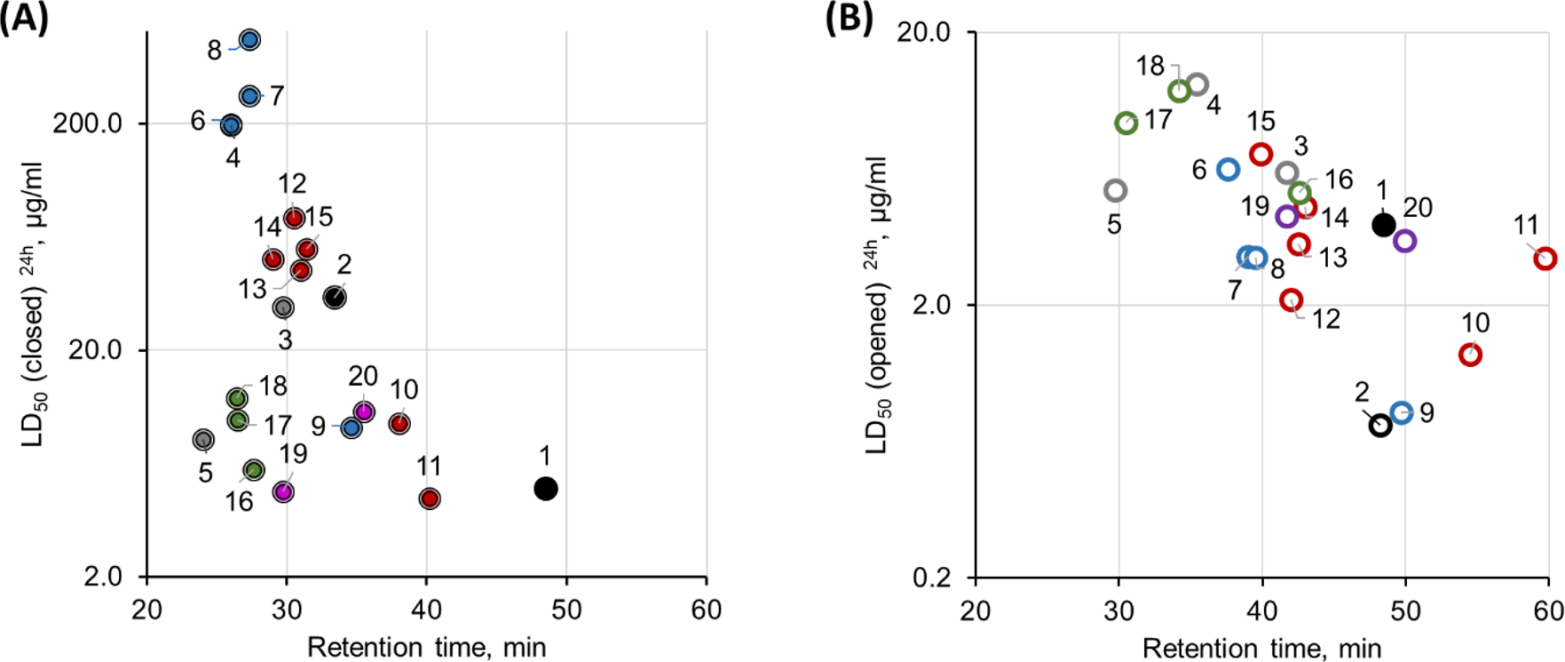
The in vivo toxicity against *D. rerio* embryos appears to be correlated with the empirical hydrophobicities of GS **1** and its photoswitchable analogues **2**–**20**. LD_50_ values are plotted against the HPLC (C_18_) retention times upon elution with a standardized linear water/acetonitrile gradient: both, for the ring-closed photoforms (A, filled circles), as well as for the ring-open photoforms (B, open circles). The compound numbers are shown next to the data points. Values for the parent peptide **1** are shown as black filled circles; the original prototype **2** is color-coded in black; the data points for peptides of series i (**3**–**5**) are shown in grey, series ii (**6**–**9**) in blue, series iii (**10**–**15**) in red, series iv (**16**–**18**) in green, and series v (**19**, **20**) in purple.

We have also analyzed the correlation between the empirical low-pH hydrophobicity of the peptides (as assessed from the retention times in reversed-phase HPLC) and toxicity against zebrafish embryos. Independent on the photoisomeric state and the type of toxicity assay, decreasing polarity of the peptides is accompanied by an increase in toxicity against zebrafish embryos (compare Figure 3A and 3B). Within the different series of peptides (the series are marked with different colors in Figure 3 for clarity) we get almost linear toxicity–hydrophobicity correlations in most cases, and all the outliers are readily explainable. For instance, in Figure 3A the peptides **19** and **20** are from series v (homodimers), compounds **16**–**18** have larger (extended) macrocycles and together with **5** possess a higher net charge and charge density than the rest of the peptides. For the most hydrophobic peptide **11** in the ring-open form, which deviates from the general trend, we observed a very low water solubility and tendency to aggregate.

**Figure 4:**
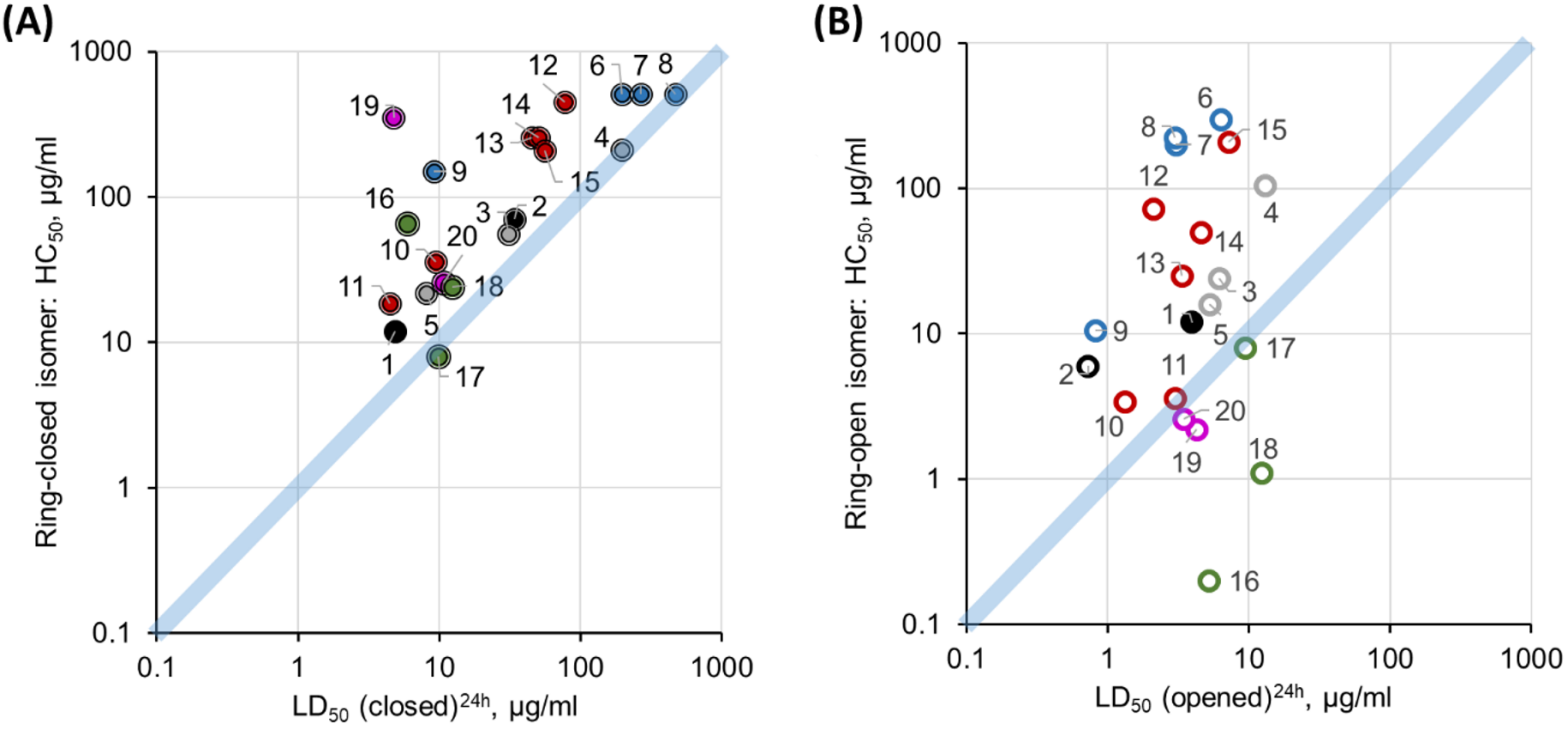
*D. rerio* embryotoxicity of GS **1** and the photoswitchable analogues **2**–**20** correlated with their in vitro hemolysis [30] for the ring-closed photoforms (A, filled circles), and for the ring-open photoforms (B, open circles). The compound numbers are shown next to the data points, and the color code is the same as in Figure 3.

As can be seen from Figure 4, the toxicity against zebrafish embryos is higher than hemolysis for the majority of the ring-open isomers and for all ring-closed photoforms. This result suggests that the lysis of red blood cells may not be the leading cause of in vivo toxicity for these membranolytic peptides.

Based on the results described above, we wondered whether the cytotoxicity against epithelial cells should be considered as an important safety aspect for applications in humans, and whether it should be monitored in preclinical evaluation of this type of chemotherapeutics. It is known, that compounds from the surrounding media are absorbed by the zebrafish mainly through the skin and gills at embryonic stages and through the digestive system during later larval stages [54], which points to epithelial cells as the immediate target and should correspond to the dermal route of administration or other through-epithelium paths in human applications. Notably, it has been shown that toxicities against zebrafish larvae may correlate with rodent inhalational toxicities [55], but to the best of our knowledge, no such comparisons are known for peptides or peptidomimetics.

**Figure 5:**
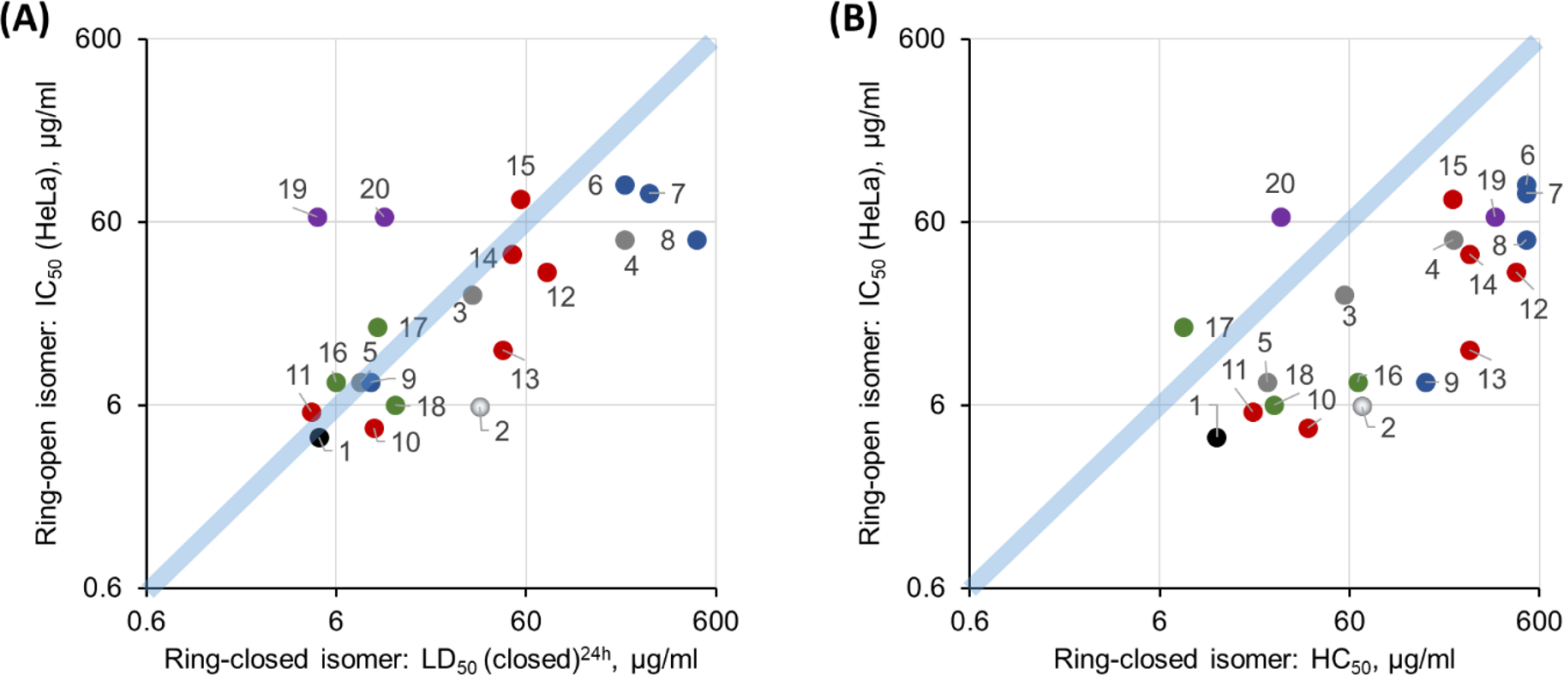
Phototherapeutic cytotoxic action against HeLa cells of GS **1** and its photoswitchable analogues **2**–**20**, correlated with the new in vivo results and earlier in vitro data. For each peptide, the HeLa cytotoxic concentrations of the ring-open photoforms (IC_50_(open)) are plotted against the corresponding in vivo toxicities to *D. rerio* embryos (A, LD_50_(closed)^24h^), or against the in vitro hemolytic activities (B, HC_50_(closed), i.e., 50% hemolysis]) of the ring-closed photoforms. The compound numbers are shown next to the data points. The in vitro data is from [30]. Values for the parent peptide **1** are marked black, and a white filling highlights the initial prototype **2**. Color codes for the remaining peptides are the same as in Figure 3.

When plotting the *D. rerio* embryotoxicity LD_50_ values of the ring-open (activated, ON) photoforms against the in vitro HeLa cytotoxicity indicators, namely against IC_50_ [30] of the ring-closed (deactivated, OFF) photoforms (Figure 5A), the data points are correlated quite well along the diagonal. This correspondence is in contrast to the comparison between hemolysis and HeLa cytotoxicity (Figure 5B) that had been reported in our earlier study, where the whole data set shows a systematic deviation off the diagonal. Our new in vivo data, thus shows a much better correlation of the toxicities between the two tested targets – epithelial cells of the zebrafish embryo and human malignant epithelial cells. Erythrocytes, on the other hand, are clearly less suitable and constitute less representative cells to predict toxic effects in humans. We can therefore expect an even lower in vivo anticancer selectivity of our peptides than we had previously judged from the in vitro data on erythrocytes and HeLa cells. Thus, a further increase of the phototherapeutic indices by compound modifications is appropriate. Amongst the most selective compounds against cancer cells, the modifications implemented in series i, ii and iii are the most effective strategies so far to enhance the phototherapeutic indices. The present evaluation identifies peptides **2**, **4**, **7**, **8**, and **13** as the best candidates in terms of the phototherapeutic index.

## Conclusion

In this study, we have synthesized 19 photoswitchable membranolytic peptides, derived from the cyclic parent gramicidin S. The two photoforms of these dithienylethene-modified peptides showed different retention times in reversed-phase HPLC, with the ring-open forms being more hydrophobic. An in vivo toxicity assay (using two approaches, giving essentially the same results) was established in order to study the in situ photoactivation of these peptides using a zebrafish embryo model. We systematically evaluated the toxicities of the two photoforms and found that the activated ring-open isomers of our peptides are more toxic than the inactivated ring-closed isomers, with up to two orders of magnitude difference. The most promising modifications of GS appear to be those where a single uncharged polar amino acid has been introduced on the hydrophilic face of the peptide.
